# Placental pathology in a pregnant woman with severe COVID-19 and successful ECMO treatment: a case report

**DOI:** 10.1186/s12884-021-04228-z

**Published:** 2021-11-10

**Authors:** Anna Rosner-Tenerowicz, Tomasz Fuchs, Aleksandra Zimmer-Stelmach, Michał Pomorski, Martyna Trzeszcz, Jacek Zwierzchowski, Mariusz Zimmer

**Affiliations:** 1 Department of Gynecology and Obstetrics, University Hospital in Wroclaw, ul. Borowska 213, 50-556 Wroclaw, Poland; 2 Division of Pathology and Clinical Cytology, University Hospital in Wroclaw, Wroclaw, Poland; 3 Department of Anaesthesiology and Intensive Care, University Hospital in Wroclaw, Wroclaw, Poland

**Keywords:** Pregnancy, COVID-19, Respiratory failure, Acute respiratory distress syndrome, ARDS, ECMO, Placental histopathology

## Abstract

**Background:**

Infection with SARS-CoV-2 during pregnancy can lead to a severe condition in the patient, which is challenging for obstetricians and anaesthesiologists. Upon severe COVID-19 and a lack of improvement after multidrug therapy and mechanical ventilation, extracorporeal membrane oxygenation (ECMO) is introduced as the last option. Such treatment is critical in women with very preterm pregnancy when each additional day of the intrauterine stay is vital for the survival of the newborn.

**Case presentation:**

We report a case of a 38-year-old woman at 27 weeks of gestation treated with multidrug therapy and ECMO. The woman was admitted to the intensive care unit (ICU) with increasing fever, cough and dyspnoea. The course of the pregnancy was uncomplicated. She was otherwise healthy. At admission, she presented with severe dyspnoea, with oxygen saturation (SpO2) of 95% on passive oxygenation, heart rate of 145/min, and blood pressure of 145/90. After confirmation of SARS-CoV-2 infection, she received steroids, remdesivir and convalescent plasma therapy. The foetus was in good condition. No signs of an intrauterine infection were visible. Due to tachypnea of 40/min and SpO2 of 90%, the woman was intubated and mechanically ventilated. Due to circulatory failure, the prothrombotic activity of the coagulation system, further saturation worsening, and poor control of sedation, she was qualified for veno-venous ECMO. An elective caesarean section was performed at 29 weeks on ECMO treatment in the ICU. A preterm female newborn was delivered with an Apgar score of 7 and a birth weight of 1440 g. The newborn had no laboratory or clinical evidence of COVID-19. The placenta showed the following pathological changes: large subchorionic haematoma, maternal vascular malperfusion, marginal cord insertion, and chorangioma.

**Conclusions:**

This case presents the successful use of ECMO in a pregnant woman with acute respiratory distress syndrome in the course of severe COVID-19. Further research is required to explain the aetiology of placental disorders (e.g., maternal vascular malperfusion lesions or thrombotic influence of COVID-19). ECMO treatment in pregnant women remains challenging; thus, it should be used with caution. Long-term assessment may help to evaluate the safety of the ECMO procedure in pregnant women.

## Background

According to the newest data, pregnant women are at increased risk for severe symptoms of COVID-19 compared with nonpregnant women [1]. The majority of cases of SARS-CoV-2 infection in pregnant women are asymptomatic or have mild influenza-like symptoms only. However, 4–6% of pregnant women with COVID-19 are admitted to the intensive care unit (ICU) due to severe outcomes of viral infection [2]. In severe cases with no improvement after multidrug therapy and mechanical ventilation, extracorporeal membrane oxygenation (ECMO) is introduced as the last option. Such treatment is critical in very preterm pregnancy when each additional day of the intrauterine stay is vital for the survival of the newborn.

## Case presentation

We present a case of a pregnant woman at 27 weeks of gestation who was successfully treated with veno-venous ECMO. Additionally, we report histopathological changes that were found in the placenta after preterm delivery.

On 02 October 2020, a 38-year-old woman at 27 weeks of gestation presented to the ICU of the Tertiary University Hospital in Wroclaw, Poland with acute pneumonia due to SARS-CoV-2 infection. On the 7th day of the illness, the patient was transferred from a second-degree hospital, where she was admitted due to increasing fever, cough and dyspnoea. She had no underlying medical history. It was her second pregnancy and second labour. Prenatal visits did not reveal any complications.

At admission, she presented with severe dyspnoea, with oxygen saturation (SpO2) of 95% on passive oxygenation, heart rate of 145 per minute, and blood pressure of 145/90. Due to high levels of inflammatory markers, the patient had been tested for viral and bacterial organisms. COVID-19 was confirmed from a nasopharyngeal swab with a polymerase chain reaction test, which was positive. The following inflammatory markers were high: WBC 23.6 × 1000/μL; CRP 223 mg/L; procalcitonin 0.81 ng/m. There were no ketones in the urine, and elevated LDH 324 U/L and troponin 11.4 pg/mL were within the normal range. According to worsening patient status, the requirement for higher doses of supplemental oxygen and high inflammatory markers, the treatment was escalated. She received remdesivir and convalescent plasma therapy. We also administered dexamethasone and azitromycin. We did not use the prone position.

We performed bedside obstetrician control to assess the foetal status. The ultrasound revealed a 1300 g foetus, which was in the 90th percentile. The placenta was localized on the posterior wall. No signs of an intrauterine infection were visible. The amniotic fluid was in a normal range. Umbilical artery Doppler and middle cerebral artery Doppler indicated good foetal conditions. The scans were performed by an experienced specialist to shorten the examination time. We did not monitor the patient with cardiotocography (CTG) due to many artefacts, which hindered our ability to obtain a high-quality trace. An obstetrical consultation was performed every day.

The next day, due to tachypnea of 40/min and SpO2 of 90% (on high flow 60 L/min, 90% O2), she was intubated and went on mechanical ventilation [pressure-controlled ventilation volume guaranteed (PCV-VG) 500; respiratory rate - 18, positive end-expiratory pressure (PEEP) – 15 cm H2O; fraction of inspired oxygen (FiO2) – 100] with propofol, fentanyl and noradrenaline sedation. Diuresis was stimulated with furosemide bolus. She was on enteral feeding. To prevent starvation ketoacidosis, the patient was fed an enteral probe with an industrial diet. We administered enteral feeding according to indirect calorimetry with Fresubin HP Energy. We supplemented vitamins C and B1. Starting at 5 days of hospitalization, parenteral nutrition was started. To reduce constipation, the patient was treated with lactulose and mannitol.

On the 4th day of hospitalization, the patient was treated with noradrenaline due to circulatory failure. A breathing multitest showed a *Haemophilus influenzae* infection; thus, antibiotic therapy was continued according to the results of the antibiogram. Azithromycin was excluded, but we used additional antibiotics, including piperacillin with tazobactam.

A prophylactic dose of anticoagulation treatment was started from the beginning of hospitalization with a low-molecular-weight heparin dose of 40 mg subcutaneously. According to rising levels of D-dimers (1.233 μg/mL), fibrinogen (9.04 g/L) and high inflammatory markers, the treatment was escalated. On the 5th day, the patient was treated with unfractionated heparin 2500 units in nebulization 4 times a day and fractionated heparin twice 40 mg subcutaneously. Because saturation worsened, the patient needed additional sedation with midazolam. The patient’s condition was stable, and we decided to assess foetal condition every 2 days.

On the 9th day of hospital stay, due to very high doses of intravenous medications and poor control of sedation, the AnaConDa system (Sedana Medical, Uppsala, Sweden) with sevoflurane was used. The patient was intubated on mechanical ventilation in bilevel mode 90% O2 and saturation 90%, with a high level of carbon dioxide. The body temperature was 39.3 °C. Because of a high level of dioxides despite high concentration oxygen therapy, the patient was qualified for veno-venous ECMO. This decision was made after consulting with a multidisciplinary team and the patient’s family. ECMO was inserted into the patient’s veins under ultrasound guidance and obstetrical control of foetal status. The flow was set at 3 L/min with heparin infusion 5/50–22 mL/h and SPO2 91% with FiO2 65%. Activated clotting time (ACT) was controlled every 3 h. CTG and ultrasound examination showed the good condition of the foetus.

On the 11th day of hospitalization, the patient was in severe but stable condition on a multidrug sedation score of 4 in the Richmond Agitation-Sedation Scale (RASS) on noradrenaline infusion. Intubation was set with ventilation Assist-Control A/C, FiO2 0.65%, PEEP 11 cmH2O, inspiratory pressure (Pinsp) 25 cm H2O and ECMO therapy in veno-venous system (SPVO2 70%, ACT 160 s). The patient had her own diuresis and was fed with a probe using Fresubin 30 mL/h and was still on antibiotic therapy (meropenem, vancomycin).

The obstetric ultrasound examination revealed suspicion of haematoma in the placenta but was difficult to confirm due to the localisation of the placenta on the posterior wall. The Doppler flow in the umbilical artery, middle cerebral artery, and ductus venosus was within proper percentiles. The CTG tracing showed a normal foetal heart rate – a baseline of 140 per minute with proper variability and the presence of accelerations. The patient received steroids a few days before caesarean section and thus met the criteria to induce foetal lung maturity. MgSO4 was used before the planned caesarean section for neuroprotection.

On 13 October 2020, an elective caesarean section was planned. Four units of packed red blood cells (PRBCs), 4 units of fresh frozen plasma (FFP) and 2 units of platelets were planned. Heparin infusion was stopped 3 h before the procedure. Caesarean section was performed in the ICU. The patient was opened from a straight central incision and closed with single sutures. A preterm female newborn was delivered at 11:15, with an Apgar score of 7 and a birth weight of 1440 g. We left the drains after caesarean section in the pouch of Douglas. The newborn had no laboratory or clinical evidence of SARS-CoV-2 infection.

The next day, the patient was in a stable condition on multidrug sedation with propofol, ketamine, fentanyl and midazolam, RASS score of 4 and the following ventilation parameters: bilevel FiO2 70%, PEEP 10 cm H2O, Pinsp 15 cmH2O, and still on veno-venous ECMO therapy. In the afternoon, heparin infusion was started with ACT 172 control. Circulation was stabilized with noradrenaline. The temperature was 36.6 °C. The patient had her own proper diuresis. We started Fresubin infusion. The gynaecological examination showed the uterus in proper tension, 1–2 fingers under the umbilicus, with postoperative bleeding in a normal range. We administered bromocriptine to stop lactation. The breasts were soft. Three days after caesarean section, tracheostomy was performed due to life indications. The postoperative course was stable. On 20 October 2020 (7 days following the procedure), the suture was removed after a caesarean section. The uterus was above the pubic symphysis, the lochia was normal, and the breasts were soft. An ultrasound revealed a haematoma starting from a wound area to 4 cm above the left side of the umbilicus. We stopped heparin infusion and transfused cryoprecipitate, fibrinogen, PRBCs, FFP and platelets.

On the 9th day after caesarean section, we performed a laparotomy because of haemodynamic instability and the high risk of haemorrhagic shock.

We evacuated two haematomas localized in the abdominal lining: one between the urinary bladder and pubic symphysis and the other from the epigastric area, reaching the left hypochondrium and descending into the left iliac region to the upper spine of the anterior iliac crest. We used sponges with fibrin and thrombin to ensure haemostasis. We left two drains, one in the abdominal cavity and the other above the fascia. The postoperative state of the patient was stable. Three days after relaparotomy, on 25 October 2020, ECMO was removed. During the next 7 days, mechanical ventilation was periodically used and finally stopped 22 days after caesarean section. The patient remained under psychiatric, neurological and intensive rehabilitation care.

On 10 November 2020, after 39 days of hospitalization, the patient was in good condition without any neurological complications and was transferred to the Obstetrics and Gynaecology Department. On 18 November 2020, 36 days after caesarean section, she was discharged home in good condition. On 27 November 2020, 46 days after caesarean section, the baby was also discharged home without any complications.

## Placental pathology and SARS-CoV-2 immunohistochemistry

**Fig. 1:**
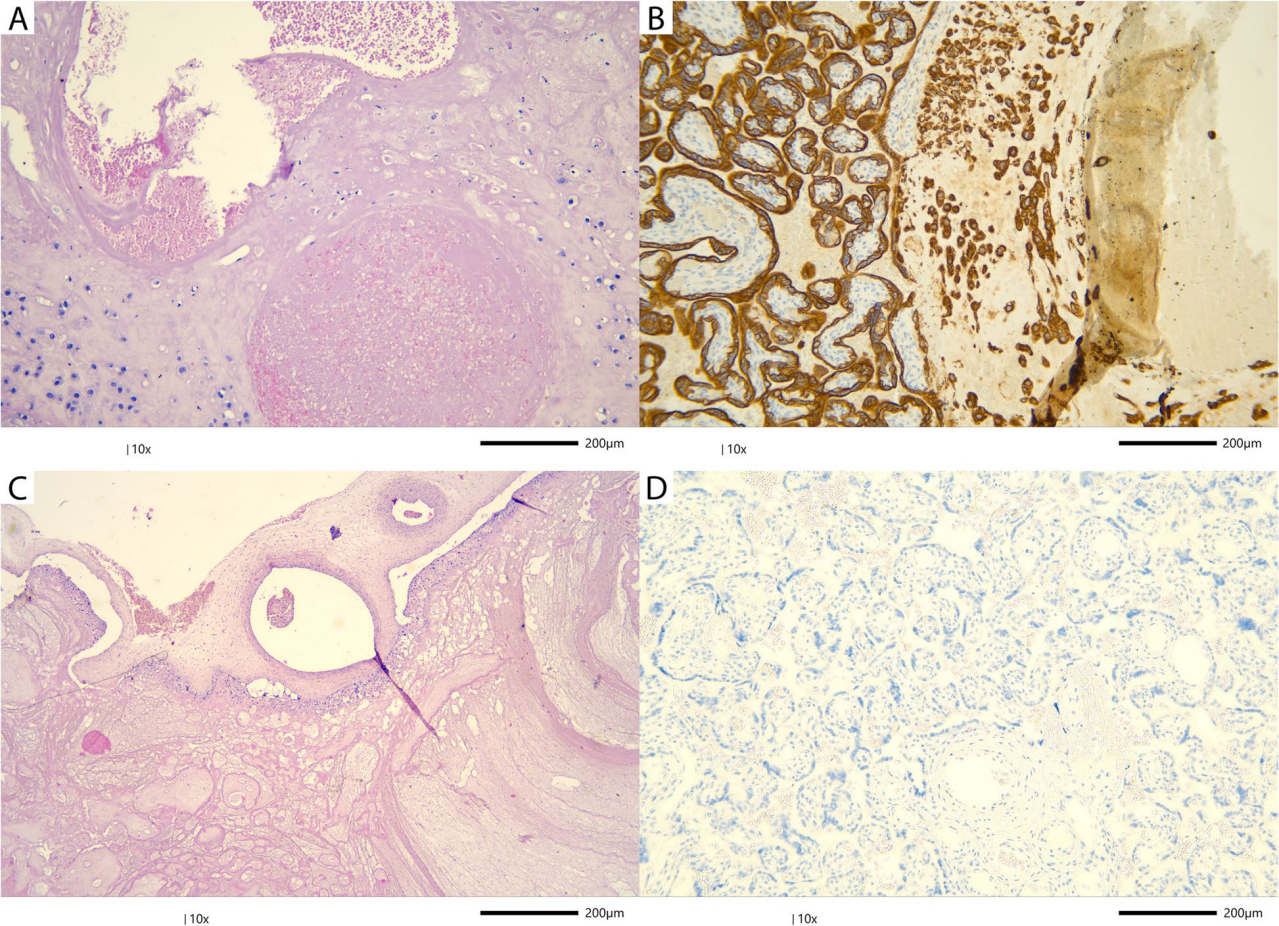
Placental pathology. **A** Decidual arteriopathy: thrombosis in spiral arteries of the basal plate. The spiral artery on the bottom is almost entirely occluded. H&E-stained section at 10X. **B** Presence of intramural endovascular trophoblasts in the third trimester. This feature is included in the spectrum of decidual arteriopathy. A few scattered trophoblast cells located in the lumen of the spiral artery are presented on the right side of the microphotograph. Trophoblast cells were highlighted by the cytokeratin immunostaining. **C** Subchorionic haematoma. Beneath the chorionic plate, the haematoma was seen on the right side together with overlying infarction on the left side. H&E-stained section at 10X. **D** Immunohistochemistry for SARS-CoV-2 in the placenta. The picture shows negative immunostaining in the placental parenchyma

For a pathological examination of placentas from SARS-CoV-2-positive mothers, a special safety procedure was introduced. Following delivery, the whole placenta was placed in 10% buffered formalin and fixed for more than 72 h before the gross pathological examination was made to avoid a potential risk of viral transmission and infection. The placental examination was performed by a qualified perinatal pathologist according to the protocol and diagnostic criteria of the Amsterdam Placental Workshop Group Consensus Statement [3]. Due to abnormal lesions identified by gross examination, the additional samples were submitted as follows: a) 2 samples of the extraplacental membranes; b) 4 cross-sections of the umbilical cord (2 for foetal and 2 for placental end); c) 12 samples from the placental parenchyma, including 9 from abnormal-appearing areas and 3 full-thickness samples from normal-appearing areas. A gross placental examination showed a large subchorionic haematoma involving 35% of the placental parenchyma, with the largest dimensions being 9.5 cm and 2.3 cm deep (Fig. 1C). At the periphery of the haematoma, the infarct was noted and involved 10–15% of the parenchyma. The fixed placenta weight was 380 g (>90th percentile at 29 weeks of gestation) and had a marginal cord insertion. Abnormal placental weight could be associated with the presence of a haematoma. The foetal-placental weight ratio was between the 50th and 75th percentiles. Thrombosis of spiral arteries in the basal plate and extravillous trophoblasts presented intraluminally were seen upon microscopic examination, and these findings are consistent with decidual arteriopathy (Fig. 1A and B). A small chorangioma was also found (1 cm in diameter). Trophoblast cells were highlighted using a PAN-CK antibody (Fig. 1B). Chronic chorionitis was identified in foetal membranes, which was highlighted using CD3 and CD8 antibodies. The SARS-CoV-2 placental immune profile was negative (Fig. 1D). SARS-CoV-2 immunohistochemistry was performed using a monoclonal antibody (GeneTex, 1A9, 1:200) at the DAKO Autostainer (DAKO Colorado, Inc., USA) according to the manufacturer’s protocol, as provided by the coordinator from the laboratory, with a negative control presented in each run. The microimages presented in Fig. 1 were acquired using the Leica DM2000 LED microscope (Leica Microsystems CMS GmbH, Germany) with the Jenoptik Gryphax NAOS camera (Progres Gryphax NAOS, Germany) in a 300dpi resolution.

## Discussion and conclusions

When all conventional treatment options fail, ECMO should be used in the critical phase of infection. In our case, we used glucocorticoids, azithromycin, remdesivir and convalescent plasma therapy. In other studies, patients were treated additionally with prone positioning, ribavirin, hydroxychloroquine, and anticytokine [4]. We wanted to show the obstetrician view on perinatal care of severe COVID-19 infection in pregnant women and noted the connections and influence of COVID-19 infection and ECMO treatment on placental changes.

The use of ECMO, as shown in the present case report, in the mid-trimester of pregnancy in a patient with COVID-19 is rare. In one of the published papers, the authors describe using ECMO in 9 pregnant women during the COVID-19 pandemic. Of these, 5 were connected to ECMO devices within 48 h postpartum, 2 during peripartum, and 2 during pregnancy. Two patients were in the third trimester of pregnancy. Ultimately, all newborns were born by caesarean section before 37 weeks of pregnancy. Although vertical transmission of COVID-19 from a mother to the foetus is possible, none of the newborns had confirmed COVID-19. In our case, the newborn was also healthy. The only neonatal complication was prematurity.

Based on the literature, the most common side effect in pregnant patients connected to ECMO was bleeding, similar to the case presented by our team. The authors reported one case of minor vaginal bleeding during ECMO and one case of the surgical procedure after ECMO decannulation for surgical site bleeding. Our patient developed bleeding after caesarean section, which resulted in a massive haematoma above the fascia requiring a relaparotomy. The authors also reported coagulation disorders as another complication. Two patients developed thrombotic complications of the membrane oxygenator with ECMO support [4]. To reduce the risk of bleeding, we should implement strict monitoring of the coagulation system by thromboelastometry while checking the level of fibrinogen and other coagulation factors. We could reduce the risk of bleeding by modifying the doses of anticoagulant treatment. According to the findings, therapy with cryoprecipitates and coagulation factors should be immediately started. Additionally, we had to remember good intraoperative haemostasis and leave the drains in the abdominal cavity as well as under the abdominal lining.

The changes observed in the placenta in the histopathological examination reflect coagulation disorders in the morphologic manifestation of a large subchorionic haematoma. When using ECMO and high doses of heparin, careful monitoring of the placenta should be carried out due to the increased risk of bleeding into the subchorionic space. Subchorionic haematomas have been associated with preterm delivery, spontaneous abortion, vaginal bleeding, midtrimester losses, intrauterine growth restriction and foetal demise. In addition, in a correlation with our case, they are more often found in maternal circulatory disorders [5].

Some identified changes related to maternal vascular malperfusion (MVM), such as infarction of placental tissue occupying up to 15% of the placenta, could have resulted from tissue compression by the haematoma. Other identified features of MVM, such as decidual arteriopathy, are consistent with other reports considering placentas from SARS-CoV-2-positive mothers, including a recently published review on the effects of COVID-19 on the placenta and pregnancy. MVM has been found in 43.2% of analysed studies in placentas of SARS-CoV-2-positive mothers, and it was the highest reported frequency [6].

The presence of chronic chorionitis of foetal membranes found in our case has ambiguous significance. Chronic chorioamnionitis could be a part of the morphologic manifestation of villitis of unknown aetiology (VUE). However, features of VUE were not found in this placenta. The significance of chronic chorionitis and its association with SARS-CoV-2 maternal infection require further study.

Vascular malperfusion and villitis of unknown aetiology were found in placentas of patients with COVID-19 in previous studies [7, 8]. Neither study found evidence of vertical transmission to the foetus, which is in line with our case, where the newborn was born healthy without vertical viral transmission.

Interestingly, in our case, we did not identify morphologic features of SARS-CoV-2 placentitis manifested as massive chronic histiocytic intervillositis with increased perivillous fibrinoid deposition, as reported recently by other authors [9]. However, it should be highlighted that SARS-CoV-2 immunoexpression in the placental parenchyma was negative in our case. Thus, it might suggest that a morphologic placental manifestation is rather unlikely. As the pandemic continues, we have begun to observe massive chronic histiocytic intervillositis as a causative agent of intrauterine foetal demise or other adverse neonatal outcomes in cases of SARS-CoV-2-positive placentas by immunostaining. This issue is the subject of another study by our group.

It remains unclear whether preterm placentas result from maternal vascular malperfusion lesions or the thrombotic influence of COVID-19 [10, 11]. Therefore, it is very important to conduct histopathological examinations of the placenta and immunological tests in newborns from mothers with confirmed COVID-19. The decision to end the pregnancy on ECMO treatment is a balance between the safety of used therapy and the risk of death due to foetal prematurity. ECMO should be used with caution in highly specialized centres under continuous obstetric supervision. Long-term assessment of future cases is crucial to evaluate the safety of the ECMO procedure in pregnant women.

## Data Availability

All data generated or analysed during this study are included in this published article.

## Abbreviations

ACT: Activated clotting time
CTG: Cardiotocography
ECMO: Extracorporeal membrane oxygenation
FiO2: Fraction of inspired oxygen
FFP: Fresh frozen plasma
ICU: Intensive care unit
PCV-VG: Pressure-controlled ventilation volume guaranteed
PEEP: Positive end-expiratory pressure
Pinsp: Inspiratory pressure
PRBCs: Packed red blood cells
RASS: Richmond Agitation-Sedation Scale
SpO2: Oxygen saturation
